# Supplementary material for: DNA modifications impact natural transformation of Acinetobacter baumannii
Source: Nucleic Acids Res. 2023 May 13;51(11):5661–77. doi: 10.1093/nar/gkad377 (PMC10287943; doi:10.1093/nar/gkad377)
Supplement: gkad377_Supplemental_File [file gkad377_supplemental_file.pdf]

Supplementary Material

DNA modifications impact natural transformation of *Acinetobacter baumannii*

Nina Vesel[1], Christian Iseli[2,3], Nicolas Guex[2,3], Alexandre Lemopoulos[1], Melanie Blokesch[1*]

[1] Laboratory of Molecular Microbiology, Global Health Institute, School of Life Sciences, Ecole Polytechnique Fédérale de Lausanne (EPFL), CH-1015 Lausanne, Switzerland

[2] Bioinformatics Competence Center, Ecole Polytechnique Fédérale de Lausanne, CH-1015 Lausanne, Switzerland

[3] Bioinformatics Competence Center, University of Lausanne, CH-1015 Lausanne, Switzerland

Supplementary Figures

A

	A118	ATCC17978	ATCC19606	AB5075	29D2	86II/2C
A118	100 / 100	45.9 / 98.7	80.8 / 97.7	96.9 / 98.7	97.0 / 99.0	97.9 / 98.7
ATCC17978		100 / 100	41.7 / 97.6	45.8 / 98.8	46.1 / 98.9	46.1/ 99.0
ATCC19606			100 / 100	79.9 / 97.6	80.4 / 97.7	80.7 / 97.8
AB5075				100 / 100	96.3 / 98.8	96.5 / 99.0
29D2					100 / 100	97.4 / 99.0
86II/2C						100 / 100

B

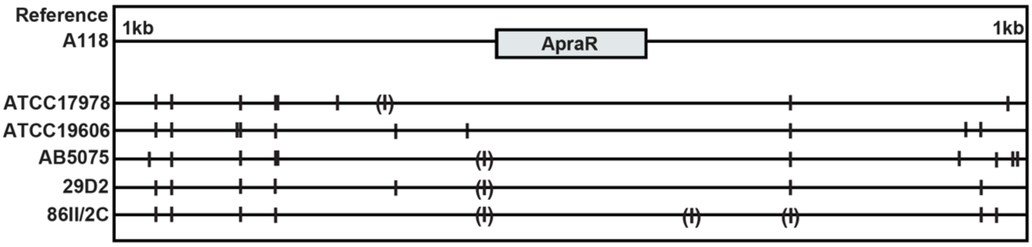

Figure S1. Sequence identity surrounding the inserted apramycin resistance cassette in different *A. baumannii* strains.

(A) Pairwise identity of DNA sequences between different *A. baumannii* strains comparing 10-kb upstream / downstream from the inserted apramycin resistance cassette. Values were calculated using the Geneious Prime software and are shown in percentage. **(B)** Alignment of DNA sequences from different *A. baumannii* strains depicting the 1-kb upstream and downstream region relative to the inserted apramycin cassette. Single nucleotide polymorphisms (SNPs) compared to the reference strain A118 are indicated by short vertical lines; if in brackets, the initial SNP was lost during the transformation-based strain construction.

A118	ATCC17978	ATCC19606	AB5075	29D2	86II2C	
H0N27_01045	H0N28_17225	H0N29_01055	OSV63_08965	OSV61_01025	OSV60_01035	class_I_SAM−dependent_methyltransferase
H0N27_01430	H0N28_16775	H0N29_01440	OSV63_08575	OSV61_01415	OSV60_01560	methyltransferase_domain−containing_protein
H0N27_01720	H0N28_16485	H0N29_01735	OSV63_08285	OSV61_01715	OSV60_01850	class_I_SAM−dependent_methyltransferase
H0N27_02900	H0N28_15060	H0N29_02925	OSV63_06870	OSV61_02900	OSV60_03060	SAM − dependent_methyltransferase
H0N27_03000	H0N28_14960	H0N29_03025	OSV63_06770	OSV61_03000	OSV60_03160	methyltransferase
H0N27_05920	H0N28_11835	H0N29_06195	OSV63_02765	OSV61_05980	OSV60_06300	class_I_SAM−dependent_methyltransferase
H0N27_09035	H0N28_08710	H0N29_09175	OSV63_18685	OSV61_09455	OSV60_09715	methyltransferase_domain−containing_protein
H0N27_10530	H0N28_06940	H0N29_10735	OSV63_17215	OSV61_10955	OSV60_11240	class_I_SAM−dependent_methyltransferase
H0N27_11860	H0N28_05480	H0N29_12680	OSV63_15670	OSV61_12340	OSV60_12655	class_I_SAM−dependent_methyltransferase
H0N27_14365	H0N28_03290	H0N29_15535	OSV63_13355	OSV61_14935	OSV60_15010	class_I_SAM−dependent_methyltransferase
H0N27_15025	H0N28_02520	H0N29_16195	OSV63_12585	OSV61_15585	OSV60_16060	class_I_SAM−dependent_methyltransferase
H0N27_15560	H0N28_01985	H0N29_16790	OSV63_12045	OSV61_16125	OSV60_16650	SAM−dependent_methyltransferase
H0N27_16335*	H0N28_01205*	H0N29_17560*	OSV63_11265*	OSV61_16905*	OSV60_17425*	DNA_adenine_methylase
H0N27_10820						DNA_adenine_methylase
H0N27_12600						DNA_adenine_methylase
	H0N28_08035					methyltransferase_domain−containing_protein
	H0N28_10790					DNA_cytosine_methyltransferase
	H0N28_14480	H0N29_03505			OSV60_03915	methyltransferase_domain−containing_protein
	H0N28_05630	H0N29_12525				DNA_cytosine_methyltransferase
		H0N29_08515				DNA_cytosine_methyltransferase
		H0N29_10075				class_I_SAM−dependent_DNA_methyltransferase
		H0N29_11830				DNA_adenine_methylase
		H0N29_12980				SAM−dependent_DNA_methyltransferase
			OSV63_06320			N−6_DNA_methylase
			OSV63_09575			DNA_cytosine_methyltransferase
			OSV63_14515	OSV61_13800		site−specific_DNA−methyltransferase
					OSV60_07345	DNA_cytosine_methyltransferase
					OSV60_07355	site−specific_DNA−methyltransferase
					OSV60_13815	class_I_SAM−dependent_DNA_methyltransferase

Figure S2. Annotated DNA methylases / methyltransferases in different *A. baumannii* strains.

The table indicates the presence (gray) or absence (white) of a selection of DNA methylases or methyltransferases found in at least one of the six studied *A. baumannii* strains (strain names are shown below the table). The locus tags of the identified methylases / methyltransferases are indicated. *, gene homologs to previously identified methylase-encoding *aamA* (1).

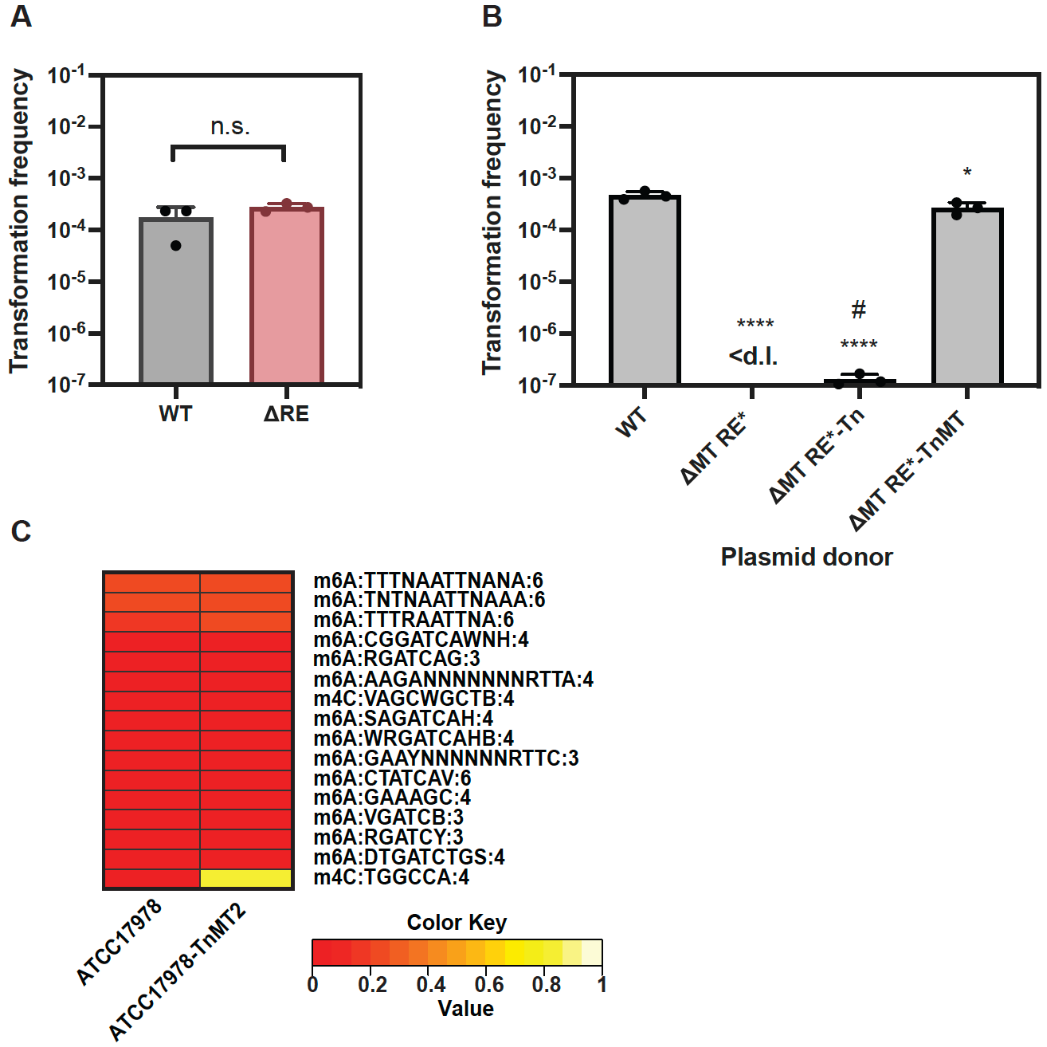

Figure S3. RMC-related MT protects transforming DNA.

(A) Transformability of strain A118 (WT) and A118ΔH0N27_10830 (ΔRE) using a PCR amplification product that encodes a rifampicin-resistant version of RpoB. **(B)** Transformability of strain A118 with plasmid pABA originating from various strains grown with 2% L-arabinose: A118 (WT), ΔH0N27_10820,H0N27_10830-K176N (ΔMT RE*), transposon control of ΔMT RE* (ΔMT RE*-Tn) or the H0N27_10820 (MT)-complemented version of ΔMT RE* (ΔMT RE*-TnMT). **(C)** Heatmap showing the abundance of certain DNA modification motifs in strain ATCC17978 and its MT2-overexpressing derivative (grown in presence of arabinose). Details as in Figure 2. The average (± SD) of three independent biological replicates is shown (A-B). <d.l., below detection limit. #, below detection limit in at least one replicate. The data was log-transformed and statistical differences were calculated using unpaired t-test (A) or a one-way ANOVA corrected for multiple comparisons with Dunnett's method (B). *, $P < 0.05$; ****, $P < 0.0001$; n.s., not significant.

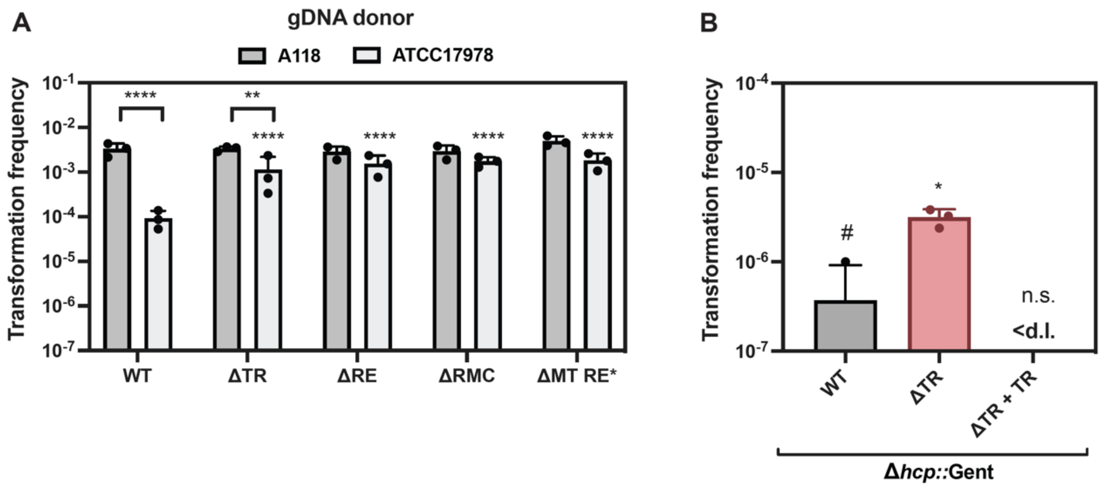

Figure S4. Transformation with foreign DNA is facilitated in the TR-deficient strain.

(A) Transformability of strain A118 and its RMC-related mutants using self (A118) and non-self (ATCC17978) genomic DNA as transforming material. Only statistically significant differences are shown. Samples for each strain after provision of different donor gDNA were compared. In addition, all strains within the specified gDNA sets were compared to the WT. **(B)** Transformability of A118 and its TR-related derivates using plasmid pABA isolated from strain ATCC17978 as transforming material. Complemented version of the TR-deficient mutant was constructed by addition of the TR gene inside *hcp* together with a gentamicin resistance cassette (ΔTR + TR). The WT and ΔTR contained the gentamicin resistance cassette inside *hcp* as a control. The mean (± SD) of three independent replicates is shown. <d.l., below detection limit. #, below detection limit in two replicates. The transformation frequency values were log-transformed and statistically analyzed using a two-way ANOVA corrected for multiple comparisons with Šidák's method (A) or an ordinary one-way ANOVA with Dunnett's correction for multiple comparisons (B). *, $P < 0.05$; **, $P < 0.01$; ****, $P < 0.0001$; n.s., not significant.

Supplementary Tables

Table S1. Bacterial strains and plasmids used in this study.

Strains or plasmids	Genotype/description*	Internal strain number	Reference (original strain and genome sequence)
A. baumannii			
A118	Wild type; Amp^R, Cm^R	MB#5144	(2-4)
ATCC17978	Wild type; Amp^R, Cm^R	MB#5143	(5)
ATCC19606	Wild type; Amp^R, Cm^R, $Strep^R$	MB#5145	(6-8)
AB5075	Wild type; Amp^R, Kan^R	MB#8577	(9,10)
29D2	Wild type; Amp^R	MB#8581	(11)
86II/2C	Wild type; Amp^R	MB#8582	(11)
A118Δhcp::aprR	A118 with *hcp* replaced by *aac(3)IV* cassette using natural transformation; Amp^R, Cm^R, Apr^R	MB#10119	This study
ATCC17978Δhcp::aprR	ATCC17978 with *hcp* replaced by *aac(3)IV* cassette using electroporation; Amp^R, Cm^R, Apr^R	MB#10120	This study
ATCC19606Δhcp::aprR	ATCC19606 with *hcp* replaced by aac(3)IV cassette using suicide plasmid pGP704Sac28-Δhcp::aprR; Amp^R, Cm^R, $Strep^R$, Apr^R	MB#10121	This study
AB5075Δhcp::aprR	AB5075 with *hcp* replaced by *aac(3)IV* cassette using natural transformation; Amp^R, Kan^R, Apr^R	MB#10122	This study
29D2Δhcp::aprR	29D2 with *hcp* replaced by *aac(3)IV* cassette using natural transformation; Amp^R, Apr^R	MB#10123	This study
86II/2CΔhcp::aprR	86II/2C with *hcp* replaced by *aac(3)IV* cassette using natural transformation; Amp^R, Apr^R	MB#10124	This study
A118 / pABA	A118 carrying pABA; Amp^R, Cm^R, Apr^R	MB#10125	This study
ATCC17978 / pABA	ATCC17978 carrying pABA; Amp^R, Cm^R, Apr^R	MB#10126	This study
ATCC19606 / pABA	ATCC19606 carrying pABA; Amp^R, Cm^R, $Strep^R$, Apr^R	MB#10127	This study
AB5075 / pABA	AB5075 carrying pABA; Amp^R, Kan^R, Apr^R	MB#10128	This study
29D2 / pABA	29D2 carrying pABA; Amp^R, Apr^R	MB#10129	This study
86II/2C / pABA	86II/2C carrying pABA; Amp^R, Apr^R	MB#10130	This study
A118ΔH0N27_10830::kanR (ΔRE)	A118 with *H0N27_10830* deleted by insertion of *aph* cassette; Amp^R, Cm^R, Kan^R	MB#9992	This study
A118ΔH0N27_10820-30::kanR (ΔRMC)	A118 with *H0N27_10820-30* deleted by insertion of *aph* cassette; Amp^R, Cm^R, Kan^R	MB#9993	This study
A118ΔH0N27_10820-30::kanR / pABA (ΔRMC / pABA)	A118ΔH0N27_10820-30::kanR carrying pABA; Amp^R, Cm^R, Kan^R, Apr^R	MB#10131	This study
A118ΔH0N27_10820-30::kanR-TnAraC (ΔRMC-Tn)	A118ΔH0N27_10820-30::kanR containing mini-Tn7-*araC* (TnAraC); Amp^R, Cm^R, Kan^R, $Gent^R$	MB#10113	This study
A118ΔH0N27_10820-30::kanR-TnAraC / pABA (ΔRMC-Tn / pABA)	A118ΔH0N27_10820-30::kanR-TnAraC carrying pABA; Amp^R, Cm^R, Kan^R, $Gent^R$, Apr^R	MB#10132	This study

A118ΔH0N27_10820-30::kanR-TnH0N27_10820 (ΔRMC-TnMT)	A118ΔH0N27_10820-30::kanR containing mini-Tn7-*araC-H0N27_10820* (TnH0N27_10820); Amp^R, Cm^R, Kan^R, Gent^R	MB#10114	This study
A118ΔH0N27_10820-30::kanR-TnH0N27_10820 / pABA (ΔRMC-TnMT / pABA)	A118ΔH0N27_10820-30::kanR TnH0N27_10820 carrying pABA; Amp^R, Cm^R, Kan^R, Gent^R, Apr^R	MB#10133	This study
A118ΔH0N27_10820::kanR-H0N27_10830-K176N (ΔMT RE*)	A118 with *H0N27_10820* deleted by insertion of *aph* cassette plus spontaneous mutation in H0N27_10830; Amp^R, Cm^R, Kan^R	MB#9995	This study
A118ΔH0N27_10820::kanR-H0N27_10830-K176N / pABA (ΔMT RE* / pABA)	A118ΔH0N27_10820::kanR H0N27_10830-K176N carrying pABA; Amp^R, Cm^R, Kan^R, Gent^R, Apr^R	MB#10134	This study
A118ΔH0N27_10820::kanR-H0N27_10830-K176N-TnAraC (ΔMT RE*-Tn)	A118ΔH0N27_10820::kanR H0N27_10830-K176N containing mini-Tn7-*araC* (TnAraC); Amp^R, Cm^R, Kan^R, Gent^R	MB#10115	This study
A118ΔH0N27_10820::kanR-H0N27_10830-K176N-TnAraC / pABA (ΔMT RE*-Tn / pABA)	A118ΔH0N27_10820::kanR H0N27_10830-K176N TnAraC carrying pABA; Amp^R, Cm^R, Kan^R, Gent^R, Apr^R	MB#10135	This study
A118ΔH0N27_10820::kanR-H0N27_10830-K176N-TnH0N27_10820 (ΔMT RE*-TnMT)	A118ΔH0N27_10820::kanR H0N27_10830-K176N containing mini-Tn7-*araC-H0N27_10820* (TnH0N27_10820); Amp^R, Cm^R, Kan^R, Gent^R	MB#10116	This study
A118ΔH0N27_10820::kanR-H0N27_10830-K176N-TnH0N27_10820 / pABA (ΔMT RE*-TnMT / pABA)	A118ΔH0N27_10820::kanR H0N27_10830-K176N-TnH0N27_10820 carrying pABA; Amp^R, Cm^R, Kan^R, Gent^R, Apr^R	MB#10136	This study
ATCC17978-TnAraC (ATCC17978-Tn)	ATCC17978 containing mini-Tn7-*araC* (TnAraC); Amp^R, Cm^R, Gent^R	MB#10117	This study
ATCC17978-TnAraC / pABA (ATCC17978-Tn / pABA)	ATCC17978-TnAraC carrying pABA; Amp^R, Cm^R, Gent^R, Apr^R	MB#10137	This study
ATCC17978-TnH0N27_10820 (ATCC17978-TnMT)	ATCC17978 containing mini-Tn7-*araC-H0N27_10820* (TnH0N27_10820); Amp^R, Cm^R, Gent^R	MB#10118	This study
ATCC17978-TnH0N27_10820 / pABA (ATCC17978 TnMT / pABA)	ATCC17978 TnH0N27_10820 carrying pABA; Amp^R, Cm^R, Gent^R, Apr^R	MB#10138	This study
ATCC17978-TnH0N27_12600 (ATCC17978-TnMT2)	ATCC17978 containing mini-Tn7-*araC-H0N27_12600* (TnH0N27_12600); Amp^R, Cm^R, Gent^R	MB#10003	This study
ATCC17978-TnH0N27_12600 / pABA (ATCC17978-TnMT2 / pABA)	ATCC17978-TnH0N27_12600 carrying pABA; Amp^R, Cm^R, Gent^R, Apr^R	MB#10139	This study
A118ΔH0N27_10825::kanR (ΔTR)	A118 with *H0N27_10825* deleted by insertion of *aph* cassette using natural transformation; Amp^R, Cm^R, Kan^R	MB#9994	This study
A118ΔH0N27_10825::kanR / pABA (ΔTR / pABA)	A118ΔH0N27_10825::kanR carrying pABA, Amp^R, Cm^R, Kan^R, Apr^R	MB#10140	This study
A118Δhcp::gentR	A118 with *hcp* replaced by *aacC1* cassette using natural transformation; Amp^R, Cm^R, Gent^R	MB#10141	This study
A118ΔH0N27_10825::kanR Δhcp::gentR	A118ΔH0N27_10825::kanR with *hcp* replaced by *aacC1* cassette using natural transformation; Amp^R, Cm^R, Gent^R	MB#10142	This study
A118ΔH0N27_10825::kanR Δhcp::H0N27_10825-gentR	A118ΔH0N27_10825::kanR with *hcp* replaced by *H0N27_10825* & *aacC1* cassette using natural transformation; Amp^R, Cm^R, Gent^R	MB#10143	This study

E. coli			
S17-1λpir	Tp^R Sm^R *recA, thi, pro, hsdR2M1*, RP4:2-Tc:Mu:Km^R, Tn7 (λpir)	MB#648	(12)
TOP10	F- *mcrA* Δ(*mrr-hsd*RMS-*mcr*BC) Φ80*lac*ZΔM15 Δ *lac*X74 *rec*A1 *ara*D139 Δ(*araleu*)7697 *gal*U *gal*K *rps*L (Strep^R) *end*A1 *nup*G	MB#4817	Invitrogen
INV110	F´ {traΔ36 proAB lacIq lacZΔM15} rpsL (Strep^R) thr leu endA thi-1 lacY galK galT ara tonA tsx dam dcm supE44 Δ(lac-proAB) Δ(mcrC-mrr)102::Tn10 (Tet^R)	MB#9225	Invitrogen
**Plasmids**			
pGP704-TnAraC	pGP704 with mini-Tn7 carrying *araC* and P_{BAD}; Amp^R, Gent^R	MB#5513	(13)
pGP704-TnH0N27_10820	pGP704 with mini-Tn7 carrying *araC* and P_{BAD}- driven *H0N27_10820*; Amp^R, Gent^R	MB#10144	This study
pGP704-TnH0N27_12600	pGP704 with mini-Tn7 carrying *araC* and P_{BAD}- driven *H0N27_12600*; Amp^R, Gent^R	MB#10145	This study
pUX-BF-13	*ori*R6K, helper plasmid with Tn7 transposition function; Amp^R	MB#457	(14)
pGP704-Sac28	suicide plasmid, ori R6K sacB; Amp^R	MB#649	(15)
pGP704-Sac28-Δhcp::aprR	pGP704Sac28 carrying a deletion within *hcp* and insertion of *aac(3)IV* cassette; Amp^R, Apr^R	MB#10146	This study
pABA	Derivative of pBAD18-Kan, with added *Acinetobacter*-specific ori (16), and replacement of *aph* cassette (Kan^R) for *aac(3)IV* cassette (Apr^R)	MB#9356	This study

*Locus tags of strain A118 and annotations are according to NCBI accession number CP059039.

Table S2. Statistics of PacBio SMRT genome sequencing and assembly for WT strains.

	ATCC17978	ATCC19606	AB5075	29D2	86II/2C
**Internal strain ID**	MB#5143	MB#5145	MB#8577	MB#8581	MB#8582
**BioSample ID**	SAMN15507625	SAMN15507635	SAMN31681936	SAMN31681934	SAMN31681935
**BioProject ID**	PRJNA645521	PRJNA645521	PRJNA900181	PRJNA900181	PRJNA900181
**GenBank accession number**	CP059041-CP059042	CP059040	CP113078-CP113079	CP113069	CP113077
**Chromosome length after circularization**	4,006,609 (+16,502 for plasmid)	3,980,852	3,995,103 (+83,610 for plasmid)	3,836,532	3,937,483
**Total size**	4,023,111	3,980,852	4,078,713	3,836,532	3,937,483
**Mean coverage**	399.24	217.27	529.45	257	403.04
**GC content**	40.90	40.20	38.91	38.91	38.90

Table S3. Statistics of PacBio SMRT genome sequencing and assembly mutant strains of A118 or ATCC17978.

	ΔTR	ΔRE	ΔRMC	ΔME RE*	ATCC17978-TnMT2
**Internal strain ID**	MB#9994	MB#9992	MB#9993	MB#9995	MB #10003
**BioSample ID**	SAMN31681938	SAMN31681939	SAMN31681940	SAMN31681941	SAMN31681942
**BioProject ID**	PRJNA900182	PRJNA900182	PRJNA900182	PRJNA900182	PRJNA900182
**GenBank accession number**	CP113070	CP113071	CP113072	CP113073	CP113074-CP113076
**Chromosome length after circularization**	3,751,367	3,750,940	3,749,418	3,750,381	4,017,814
**Total genome size**	3,751,367	3,750,940	3,749,418	3,750,381	4,042,525
**Mean coverage**	455.43	465.99	342.69	468.94	420.03
**GC content**	39.43	39.49	39.40	39.42	39.34

Table S4. Characteristics of *A. baumannii* strains.

Strains name	Year of isolation	Country of isolation	Host	Sample type	Naturally transformable	References
A118	1995	Argentina	Human	Blood	yes	(2,3)
ATCC17978	1951	?	Human	Spinal meningitis	no	(5,17)
ATCC19606	1948	US	Human	Urine	no	(6-8)
29D2	2014	Poland	White stork	Nestling; choana	yes	(11)
86II/2C	2013	Poland	White stork	Nestling; choana	yes	(11)
AB5075	2008	US	Human	Tibia/ osteomyelitis	yes	(9,10)

Table S5. Number of identified recognition sites in analyzed *A. baumannii* genomes, plasmids, and PCR fragments.

Epigenetic mark	A118	ATCC 17978	ATCC 19606	AB5075	29D2	86II/2C	plasmid pABA	PCR Δ*hcp*:: aprR	PCR *rpoB**
unk:CNNNGTRTAVCR:6	143	149	160	152	148	155	0	0	0
unk:CGGANBAVCR:4	192	222	219	205	203	205	2	1	0
unk:TTTATAVCA:1	177	173	176	191	175	173	1	0	1
unk:CGGTGTNTY:4	144	159	172	154	152	170	0	0	0
m6A:TTTRAATTNA:6	519	578	550	545	532	526	0	1	0
m6A:TNTNAATTNAAA:6	374	386	365	366	356	364	0	2	0
m6A:TAAYNNNNNNNTCTT:3	567	600	581	588	577	585	2	0	1
m6A:CTATCAV:6	1096	1214	1202	1217	1129	1156	3	2	2
m6A:AAGANNNNNNNRTTA:4	567	600	581	588	577	585	2	0	1
unk:GCGGTAMYD:3	313	327	322	327	306	330	1	0	0
m6A:RGATCY:3	2842	3404	3150	3294	3090	3080	11	4 (*)	1 (#)
unk:TGGCCA:4	1264	1398	1374	1418	1352	1316	0	0	0
m6A:GAAYNNNNNNRTTC:3	518	574	540	554	518	512	0	1	1
m4C:VAGCWGCTB:4	1414	1456	1508	1528	1422	1408	2	0	2
m6A:GAAAGC:4	2131	2284	2306	2320	2175	2257	6	1	2
m6A:CTGATCY:4	744	822	832	805	751	787	3	1	0
m6A:DTGATCTGS:4	193	219	215	227	218	216	1	1	0
m6A:SAGATCAH:4	557	647	619	633	595	619	2	1	0
m6A:CGGATCAWNH:4	177	170	172	158	187	183	2	1	0
m6A:WRGATCAHB:4	1376	1488	1471	1496	1385	1462	1	2	0
m6A:RGATCAG:3	744	822	832	805	751	787	3	1	0
m6A:VGATCB:3	6288	7210	6840	7178	6658	6688	15	8	3
m6A:TTTNAATTNANA:6	374	386	365	366	356	364	0	2	0

(*) Start of the RGATCY sites are located at -395 bp, -115 bp, -103 bp, and +727 bp relative to *aprR*'s start codon.
(#) Start of the RGATCY site is located 1968 bp downstream the Rif^{R}-causing SNP in *rpoB*.

Table S6. Number of modified versus identified motifs in the indicated *A. baumannii* strains. (supporting table for Fig. 2A)

	ATCC19606	ATCC17978	A118
unk:CNNNGTRTAVCR:6	49 / 160 (0.31)	85 / 149 (0.57)	23 / 143 (0.16)
unk:CGGANBAVCR:4	61 / 219 (0.28)	103 / 222 (0.46)	28 / 192 (0.15)
unk:TTTATAVCA:1	47 / 176 (0.27)	59 / 173 (0.34)	24 / 177 (0.14)
unk:CGGTGTNTY:4	34 / 172 (0.20)	54 / 159 (0.34)	18 / 144 (0.13)
m6A:TTTRAATTNA:6	188 / 550 (0.34)	85 / 578 (0.15)	61 / 519 (0.12)
m6A:TNTNAATTNAAA:6	90 / 365 (0.25)	70 / 386 (0.18)	58 / 374 (0.16)
m6A:TAAYNNNNNNNTCTT:3	581 / 581 (1.00)	0 / 600 (0.00)	0 / 567 (0.00)
m6A:CTATCAV:6	1202 / 1202 (1.00)	2 / 1214 (0.00)	0 / 1096 (0.00)
m6A:AAGANNNNNNNRTTA:4	581 / 581 (1.00)	9 / 600 (0.02)	14 / 567 (0.02)
unk:GCGGTAMYD:3	187 / 322 (0.58)	274 / 327 (0.84)	142 / 313 (0.45)
m6A:RGATCY:3	12 / 3150 (0.00)	0 / 3404 (0.00)	2838 / 2842 (1.00)
unk:TGGCCA:4	1 / 1374 (0.00)	3 / 1398 (0.00)	1011 / 1264 (0.80)
m6A:GAAYNNNNNNRTTC:3	3 / 540 (0.01)	3 / 574 (0.01)	1 / 518 (0.00)
m4C:VAGCWGCTB:4	3 / 1508 (0.00)	12 / 1456 (0.01)	1 / 1414 (0.00)
m6A:GAAAGC:4	0 / 2306 (0.00)	3 / 2284 (0.00)	0 / 2131 (0.00)

	86II/2C	29D2	AB5075
unk:CNNNGTRTAVCR:6	6 / 155 (0.04)	2 / 148 (0.01)	2 / 152 (0.01)
unk:CGGANBAVCR:4	1 / 205 (0.00)	0 / 203 (0.00)	0 / 205 (0.00)
unk:TTTATAVCA:1	1 / 173 (0.01)	0 / 175 (0.00)	0 / 191 (0.00)
unk:CGGTGTNTY:4	2 / 170 (0.01)	2 / 152 (0.01)	1 / 154 (0.01)
m6A:TTTRAATTNA:6	184 / 526 (0.35)	197 / 532 (0.37)	60 / 545 (0.11)
m6A:TNTNAATTNAAA:6	93 / 364 (0.26)	89 / 356 (0.25)	53 / 366 (0.14)
m6A:TAAYNNNNNNNTCTT:3	1 / 585 (0.00)	0 / 577 (0.00)	1 / 588 (0.00)
m6A:CTATCAV:6	0 / 1156 (0.00)	1 / 1129 (0.00)	0 / 1217 (0.00)
m6A:AAGANNNNNNNRTTA:4	0 / 585 (0.00)	3 / 577 (0.01)	4 / 588 (0.01)
unk:GCGGTAMYD:3	5 / 330 (0.02)	9 / 306 (0.03)	9 / 327 (0.03)
m6A:RGATCY:3	1 / 3080 (0.00)	0 / 3090 (0.00)	5 / 3294 (0.00)
unk:TGGCCA:4	11 / 1316 (0.01)	13 / 1352 (0.01)	22 / 1418 (0.02)
m6A:GAAYNNNNNNRTTC:3	512 / 512 (1.00)	4 / 518 (0.01)	2 / 554 (0.00)
m4C:VAGCWGCTB:4	1158 / 1408 (0.82)	32 / 1422 (0.02)	59 / 1528 (0.04)
m6A:GAAAGC:4	1 / 2257 (0.00)	2175 / 2175 (1.00)	2320 / 2320 (1.00)

Table S7. Number of modified versus identified motifs in the indicated *A. baumannii* strains. (supporting table for Fig. S3C)

	ATCC17978	ATCC17978-TnMT2
m6A:TTTNAATTNANA:6	68 / 386 (0.18)	75 / 386 (0.19)
m6A:TNTNAATTNAAA:6	70 / 386 (0.18)	72 / 386 (0.19)
m6A:TTTRAATTNA:6	85 / 578 (0.15)	104 / 579 (0.18)
m6A:CGGATCAWNH:4	2 / 170 (0.01)	0 / 170 (0.00)
m6A:RGATCAG:3	9 / 822 (0.01)	0 / 832 (0.00)
m6A:AAGANNNNNNNRTTA:4	9 / 600 (0.02)	4 / 600 (0.01)
m4C:VAGCWGCTB:4	12 / 1456 (0.01)	17 / 1468 (0.01)
m6A:SAGATCAH:4	2 / 647 (0.00)	1 / 652 (0.00)
m6A:WRGATCAHB:4	5 / 1488 (0.00)	1 / 1502 (0.00)
m6A:GAAYNNNNNNRTTC:3	3 / 574 (0.01)	1 / 572 (0.00)
m6A:CTATCAV:6	2 / 1214 (0.00)	0 / 1221 (0.00)
m6A:GAAAGC:4	3 / 2284 (0.00)	0 / 2290 (0.00)
m6A:VGATCB:3	7 / 7210 (0.00)	1973101 (0.00)
m6A:RGATCY:3	0 / 3404 (0.00)	571241 (0.00)
m6A:DTGATCTGS:4	0 / 219 (0.00)	1 / 219 (0.00)
m4C:TGGCCA:4	3 / 1398 (0.00)	985 / 1400 (0.70)

Table S8. Number of modified versus identified motifs in the indicated *A. baumannii* strains. (supporting table for Fig. 4F)

	A118 (reSEQ)	∆RE	∆MT RE*	∆RMC
m6A:CTGATCY:4	14 / 744 (0.02)	1 / 745 (0.00)	0 / 745 (0.00)	0 / 745 (0.00)
m6A:SAGATCAH:4	22 / 557 (0.04)	5 / 557 (0.01)	0 / 558 (0.00)	0 / 556 (0.00)
m6A:CGGATCAWNH:4	1 / 177 (0.01)	0 / 177 (0.00)	0 / 177 (0.00)	0 / 177 (0.00)
m6A:WRGATCAHB:4	3 / 1376 (0.00)	1 / 1376 (0.00)	0 / 1376 (0.00)	0 / 1376 (0.00)
m6A:RGATCAG:3	170 / 744 (0.23)	42 / 745 (0.06)	0 / 745 (0.00)	0 / 745 (0.00)
m6A:VGATCB:3	2978 / 6288 (0.47)	2861 / 6294 (0.45)	1604178 (0.00)	3 / 6292 (0.00)
m6A:TTTNAATTNANA:6	98 / 374 (0.26)	97 / 374 (0.26)	96 / 374 (0.26)	97 / 374 (0.26)
m6A:TNTNAATTNAAA:6	95 / 374 (0.25)	95 / 374 (0.25)	96 / 374 (0.26)	94 / 374 (0.25)
m6A:TTTRAATTNA:6	214 / 519 (0.41)	202 / 518 (0.39)	202 / 519 (0.39)	213 / 518 (0.41)
unk:TTTATAVCA:1	1 / 177 (0.01)	1 / 177 (0.01)	0 / 177 (0.00)	0 / 177 (0.00)
unk:CGGTGTNTY:4	0 / 144 (0.00)	0 / 144 (0.00)	1 / 144 (0.01)	0 / 144 (0.00)
unk:CNNNGTRTAVCR:6	2 / 143 (0.01)	0 / 143 (0.00)	2 / 143 (0.01)	1 / 143 (0.01)
unk:CGGANBAVCR:4	0 / 192 (0.00)	1 / 192 (0.01)	0 / 192 (0.00)	0 / 192 (0.00)
m6A:AAGANNNNNNNRTTA:4	9 / 567 (0.02)	7 / 567 (0.01)	0 / 567 (0.00)	1 / 567 (0.00)
m6A:GAAYNNNNNNRTTC:3	4 / 518 (0.01)	1 / 520 (0.00)	3 / 520 (0.01)	4 / 520 (0.01)
m4C:TGGCCA:4	1231 / 1264 (0.97)	1219 / 1264 (0.96)	1202 / 1262 (0.95)	1211 / 1262 (0.96)
m6A:RGATCY:3	2839 / 2842 (1.00)	2839 / 2844 (1.00)	0 / 2842 (0.00)	1 / 2842 (0.00)

Table S9. BLAST results of the RMC genomic region against NCBI's RefSeq Genome Database.

Description	Scientific Name	Max Score	Total Score	Query Cover	E value	Per. ident	Acc. Len	Accession
***Acinetobacter baumannii* strain A118 chromosome, complete genome (input query)**	*Acinetobacter baumannii*	3991	3991	100%	0.0	100.00	3750370	NZ_CP059039.1
***Acinetobacter baumannii* strain A118F NODE_11_length_136216_cov_13.6381, whole genome shotgun sequence**	*Acinetobacter baumannii*	3991	3991	100%	0.0	100.00	136216	NZ_VCCO01000013.1
***Acinetobacter baumannii* strain Ab42 NODE_1_length_802719_cov_1.259005, whole genome shotgun sequence**	*Acinetobacter baumannii*	3991	3991	100%	0.0	100.00	802719	NZ_JAAZUE010000001.1
***Acinetobacter baumannii* strain ARLG-1772 PR391_NODE_5.ctg_1, whole genome shotgun sequence**	*Acinetobacter baumannii*	3980	3980	100%	0.0	99.91	245745	NZ_NGEY01000012.1
***Acinetobacter baumannii* UH6507 ctgN19, whole genome shotgun sequence**	*Acinetobacter baumannii* UH6507	3980	3980	100%	0.0	99.91	313343	NZ_AYFK01000017.1
***Acinetobacter oleivorans* strain KCJK7897 NODE_3_length_378121_cov_27.2151_ID_5, whole genome shotgun sequence**	*Acinetobacter oleivorans*	3105	3105	100%	0.0	92.60	378121	NZ_QAYN01000006.1
***Acinetobacter baumannii* strain MRSN32076 MRSN32076_MRSN32076_contig00061, whole genome shotgun sequence**	*Acinetobacter baumannii*	2850	2850	100%	0.0	90.47	32390	NZ_VHFM01000061.1
***Acinetobacter baumannii* NIPH 60 acLrl-supercont1.29, whole genome shotgun sequence**	*Acinetobacter baumannii* NIPH 60	2850	2850	100%	0.0	90.47	494539	NZ_KB849502.1
***Acinetobacter baumannii* strain ABBL038 contig-37, whole genome shotgun sequence**	*Acinetobacter baumannii*	2844	2844	100%	0.0	90.43	95751	NZ_LLDS01000058.1
***Acinetobacter baumannii* strain ABBL110 contig-3000004, whole genome shotgun sequence**	*Acinetobacter baumannii*	1701	1701	57%	0.0	91.27	8035	NZ_LLHI01000148.1
***Acinetobacter tandoii* strain SC36 contig39, whole genome shotgun sequence**	*Acinetobacter tandoii*	1107	1107	97%	0.0	76.48	11215	NZ_LBNL01000039.1
***Acinetobacter* sp. YH12083 NODE_27_length_6361_cov_74.148736, whole genome shotgun sequence**	*Acinetobacter* sp. YH12083	1075	1075	100%	0.0	76.03	6361	NZ_VPAD01000030.1
***Acinetobacter* sp. YH12133 NODE_29_length_6480_cov_156.757595, whole genome shotgun sequence**	*Acinetobacter* sp. YH12133	1075	1075	100%	0.0	76.03	6480	NZ_VPBS01000029.1
***Acinetobacter* sp. YH12139 NODE_29_length_6480_cov_184.474107, whole genome shotgun sequence**	*Acinetobacter* sp. YH12139	1075	1075	100%	0.0	76.03	6480	NZ_VPBY01000029.1
***Acinetobacter* sp. YH12132 NODE_29_length_6480_cov_145.652133, whole genome shotgun sequence**	*Acinetobacter* sp. YH12132	1075	1075	100%	0.0	76.03	6480	NZ_VPBR01000029.1
***Acinetobacter indicus* strain AI31 NODE_58_length_8886_cov_1445.717675, whole genome shotgun sequence**	*Acinetobacter indicus*	1072	1072	100%	0.0	75.98	8886	NZ_JAAZRK010000058.1
***Acinetobacter indicus* strain AI29 NODE_57_length_8886_cov_817.336361, whole genome shotgun sequence**	*Acinetobacter indicus*	1072	1072	100%	0.0	75.99	8886	NZ_JAAZRM010000057.1
***Acinetobacter* sp. YH12046 NODE_51_length_8267_cov_43.768796, whole genome shotgun sequence**	*Acinetobacter* sp. YH12046	1072	1072	100%	0.0	75.98	8267	NZ_VOZF01000052.1
***Acinetobacter* sp. YH12091 NODE_34_length_8267_cov_66.187715, whole genome shotgun sequence**	*Acinetobacter* sp. YH12091	1072	1072	100%	0.0	75.99	8267	NZ_VPAJ01000034.1

***Acinetobacter* sp. YH12112 NODE_62_length_8267_cov_98.356880, whole genome shotgun sequence**	*Acinetobacter* sp. YH12112	1072	1072	100%	0.0	75.99	8267	NZ_VPBA01000063.1
***Acinetobacter* sp. YH12090 NODE_35_length_8267_cov_103.266830, whole genome shotgun sequence**	*Acinetobacter* sp. YH12090	1072	1072	100%	0.0	75.98	8267	NZ_VPAI01000035.1
***Acinetobacter* sp. YH1901152 NODE_54_length_8267_cov_61.999017, whole genome shotgun sequence**	*Acinetobacter* sp. YH1901152	1072	1072	100%	0.0	75.99	8267	NZ_VPFA01000056.1
***Acinetobacter indicus* strain AI21 NODE_57_length_6209_cov_1859.771200, whole genome shotgun sequence**	*Acinetobacter indicus*	1018	1018	98%	0.0	75.73	6209	NZ_JAAZRU010000057.1
***Acinetobacter* sp. ANC 5347 Contig37, whole genome shotgun sequence**	*Acinetobacter* sp. ANC 5347	1000	1000	100%	0.0	75.48	6481	NZ_PGOZ01000037.1
***Acinetobacter lwoffii* strain VE243-2 contig00045, whole genome shotgun sequence**	*Acinetobacter lwoffii*	929	929	84%	0.0	76.17	6909	NZ_JAMXXR010000045.1
***Acinetobacter* sp. YH12066 NODE_59_length_13185_cov_94.117016, whole genome shotgun sequence**	*Acinetobacter* sp. YH12066	854	854	83%	0.0	75.53	13185	NZ_VOZQ01000059.1
***Acinetobacter* sp. TG19627 A_sp_TG19627_7, whole genome shotgun sequence**	*Acinetobacter* sp. TG19627	843	843	83%	0.0	75.42	7121	NZ_AMJM01000006.1
***Acinetobacter genomosp.* 16BJ strain CIP 70.18 acLso-supercont1.5, whole genome shotgun sequence**	*Acinetobacter genomosp.* 16BJ	843	843	83%	0.0	75.42	7141	NZ_KB850080.1
***Acinetobacter* sp. YH16032 NODE_53_length_7154_cov_1106.629145, whole genome shotgun sequence**	*Acinetobacter* sp. YH16032	817	817	69%	0.0	76.76	7154	NZ_VPEE01000056.1
***Acinetobacter* sp. YH12105 NODE_37_length_6292_cov_57.114842, whole genome shotgun sequence**	*Acinetobacter* sp. YH12105	809	809	83%	0.0	75.24	6292	NZ_VPAU01000038.1
***Acinetobacter baumannii* strain ABBL110 contig-69, whole genome shotgun sequence**	*Acinetobacter baumannii*	643	643	22%	1E-180	90.39	14279	NZ_LLHI01000265.1
***Acinetobacter* sp. YH01025 NODE_24_length_6480_cov_287.777743, whole genome shotgun sequence**	*Acinetobacter* sp. YH01025	505	505	37%	6E-139	78.04	6480	NZ_VOYR01000024.1

Table S10. Number of modified versus identified motifs in the indicated *A. baumannii* strains. (supporting table for Fig. 5B)

	A118 (reSEQ)	∆TR
m6A:CTGATCY:4	14 / 744 (0.02)	715 / 745 (0.96)
m6A:DTGATCTGS:4	0 / 193 (0.00)	184 / 193 (0.95)
m6A:SAGATCAH:4	22 / 557 (0.04)	518 / 557 (0.93)
m6A:CGGATCAWNH:4	1 / 177 (0.01)	154 / 177 (0.87)
m6A:WRGATCAHB:4	3 / 1376 (0.00)	972 / 1376 (0.71)
m6A:RGATCAG:3	170 / 744 (0.23)	736 / 745 (0.99)
m6A:VGATCB:3	2978 / 6288 (0.47)	5532 / 6294 (0.88)
m6A:TTTNAATTNANA:6	96 / 374 (0.26)	97 / 372 (0.26)
m6A:TNTNAATTNAAA:6	95 / 374 (0.25)	95 / 372 (0.26)
m6A:TTTRAATTNA:6	214 / 519 (0.41)	216 / 517 (0.42)
unk:TTTATAVCA:1	1 / 177 (0.01)	1 / 177 (0.01)
unk:CNNNGTRTAVCR:6	2 / 143 (0.01)	2 / 143 (0.01)
unk:CGGANBAVCR:4	0 / 192 (0.00)	19 / 192 (0.10)
m6A:AAGANNNNNNNRTTA:4	9 / 567 (0.02)	20 / 567 (0.04)
m4C:VAGCWGCTB:4	16 / 1414 (0.01)	13 / 1414 (0.01)
m6A:GAAYNNNNNNRTTC:3	4 / 518 (0.01)	3 / 520 (0.01)
unk:GCGGTAMYD:3	0 / 313 (0.00)	1 / 313 (0.00)
m4C:TGGCCA:4	1231 / 1264 (0.97)	1240 / 1264 (0.98)
m6A:RGATCY:3	2839 / 2842 (1.00)	2844 / 2844 (1.00)

Supplementary References

1. Blaschke, U., Suwono, B., Zafari, S., Ebersberger, I., Skiebe, E., Jeffries, C.M., Svergun, D.I. and Wilharm, G. (2018) Recombinant production of A1S_0222 from *Acinetobacter baumannii* ATCC 17978 and confirmation of its DNA-(adenine N6)-methyltransferase activity. *Protein Expr. Purif.*, **151**, 78-85.
2. Traglia, G.M., Chua, K., Centron, D., Tolmasky, M.E. and Ramirez, M.S. (2014) Whole-genome sequence analysis of the naturally competent *Acinetobacter baumannii* clinical isolate A118. *Genome Biol. Evol.*, **6**, 2235-2239.
3. Ramirez, M.S., Don, M., Merkier, A.K., Bistue, A.J., Zorreguieta, A., Centron, D. and Tolmasky, M.E. (2010) Naturally competent *Acinetobacter baumannii* clinical isolate as a convenient model for genetic studies. *J. Clin. Microbiol.*, **48**, 1488-1490.
4. Vesel, N. and Blokesch, M. (2021) Pilus Production in *Acinetobacter baumannii* Is Growth Phase Dependent and Essential for Natural Transformation. *J. Bacteriol.*, **203**, e00034-21.
5. Adams, M.D., Goglin, K., Molyneaux, N., Hujer, K.M., Lavender, H., Jamison, J.J., MacDonald, I.J., Martin, K.M., Russo, T., Campagnari, A.A. *et al.* (2008) Comparative genome sequence analysis of multidrug-resistant *Acinetobacter baumannii*. *J. Bacteriol.*, **190**, 8053-8064.
6. Zhu, Y., Lu, J., Zhao, J., Zhang, X., Yu, H.H., Velkov, T. and Li, J. (2020) Complete genome sequence and genome-scale metabolic modelling of *Acinetobacter baumanni*i type strain ATCC 19606. *Int. J. Med. Microbiol.*, **310**, 151412.
7. Hamidian, M., Blasco, L., Tillman, L.N., To, J., Tomas, M. and Myers, G.S.A. (2020) Analysis of Complete Genome Sequence of *Acinetobacter baumannii* Strain ATCC 19606 Reveals Novel Mobile Genetic Elements and Novel Prophage. *Microorganisms*, **8**, 1851.
8. Tsubouchi, T., Suzuki, M., Niki, M., Oinuma, K.I., Niki, M., Kakeya, H. and Kaneko, Y. (2020) Complete Genome Sequence of *Acinetobacter baumannii* ATCC 19606(T), a Model Strain of Pathogenic Bacteria Causing Nosocomial Infection. *Microbiol. Resour. Announc.*, **9**, e00289-20.
9. Zurawski, D.V., Thompson, M.G., McQueary, C.N., Matalka, M.N., Sahl, J.W., Craft, D.W. and Rasko, D.A. (2012) Genome sequences of four divergent multidrug-resistant *Acinetobacter baumannii* strains isolated from patients with sepsis or osteomyelitis. *J. Bacteriol.*, **194**, 1619-1620.
10. Jacobs, A.C., Thompson, M.G., Black, C.C., Kessler, J.L., Clark, L.P., McQueary, C.N., Gancz, H.Y., Corey, B.W., Moon, J.K., Si, Y. *et al.* (2014) AB5075, a Highly Virulent Isolate of *Acinetobacter baumannii,* as a Model Strain for the Evaluation of Pathogenesis and Antimicrobial Treatments. *mBio*, **5**, e01076-14.
11. Wilharm, G., Skiebe, E., Higgins, P.G., Poppel, M.T., Blaschke, U., Leser, S., Heider, C., Heindorf, M., Brauner, P., Jackel, U. *et al.* (2017) Relatedness of wildlife and livestock avian isolates of the nosocomial pathogen *Acinetobacter baumannii* to lineages spread in hospitals worldwide. *Environ. Microbiol.*, **19**, 4349-4364.
12. Simon, R., Priefer, U. and Pühler, A. (1983) A broad host range mobilization system for *in vivo* genetic engineering: transposon mutagenesis in Gram negative bacteria. *Nat. Biotechnol.*, **1**, 784-791.
13. Adams, D.W., Stutzmann, S., Stoudmann, C. and Blokesch, M. (2019) DNA-uptake pili of *Vibrio cholerae* are required for chitin colonization and capable of kin recognition via sequence-specific self-interaction. *Nat. Microbiol.*, **4**, 1545-1557.
14. Bao, Y., Lies, D.P., Fu, H. and Roberts, G.P. (1991) An improved Tn*7*-based system for the single-copy insertion of cloned genes into chromosomes of Gram-negative bacteria. *Gene*, **109**, 167-168.
15. Meibom, K.L., Li, X.B., Nielsen, A.T., Wu, C.Y., Roseman, S. and Schoolnik, G.K. (2004) The *Vibrio cholerae* chitin utilization program. *Proc. Natl. Acad. Sci. USA*, **101**, 2524-2529.
16. Choi, A.H., Slamti, L., Avci, F.Y., Pier, G.B. and Maira-Litran, T. (2009) The *pgaABCD* locus of *Acinetobacter baumannii* encodes the production of poly-beta-1-6-N-acetylglucosamine, which is critical for biofilm formation. *J. Bacteriol.*, **191**, 5953-5963.
17. Smith, M.G., Gianoulis, T.A., Pukatzki, S., Mekalanos, J.J., Ornston, L.N., Gerstein, M. and Snyder, M. (2007) New insights into *Acinetobacter baumannii* pathogenesis revealed by high-density pyrosequencing and transposon mutagenesis. *Genes Dev.*, **21**, 601-614.
